# Akuter ischämischer Schlaganfall und erhöhter Troponinwert – Update des Mannheimer Algorithmus

**DOI:** 10.1007/s00108-024-01719-x

**Published:** 2024-06-03

**Authors:** Louisa Becker, Angelika Alonso, Mathieu Kruska, Stefan Baumann, Niklas Grassl, Hendrik Lesch, Philipp Eisele, Tina Sieburg, Michael Behnes, Tobias Schupp, Hany Kayed, Michael Platten, Daniel Duerschmied, Kristina Szabo, Ibrahim Akin, Christian Fastner

**Affiliations:** 1 Neurologische Klinik, Universitätsmedizin Mannheim (UMM) und Mannheim Center for Translational Neurosciences (MCTN), Medizinische Fakultät Mannheim, Universität Heidelberg, Mannheim, Deutschland; 2 I. Medizinische Klinik, Schwerpunkte: Kardiologie, Angiologie, Hämostaseologie und Internistische Intensivmedizin, Universitätsmedizin Mannheim (UMM), Medizinische Fakultät Mannheim, Universität Heidelberg, European Center for AngioScience (ECAS) und Deutsches Zentrum für Herz-Kreislauf-Forschung (DZHK), Standort Heidelberg/Mannheim, Theodor-Kutzer-Ufer 1–3, 68167 Mannheim, Deutschland; 3 Innere Medizin II, Abteilung für Kardiologie, Kreiskrankenhaus Bergstraße, Heppenheim, Deutschland; 4 Klinik für Radiologie und Nuklearmedizin, Universitätsmedizin Mannheim (UMM), Medizinische Fakultät Mannheim, Universität Heidelberg, Mannheim, Deutschland

**Keywords:** Akuter Myokardschaden, Chronischer Myokardschaden, Myokardinfarkt, Schlaganfallinduzierter Herzschaden, Computertomographie-Koronarangiographie, Acute myocardial injury, Chronic myocardial injury, Myocardial infarction, Stroke-induced heart injury, Coronary computed tomography angiography

## Abstract

Bei etwa der Hälfte aller Patienten mit akutem ischämischem Schlaganfall (AIS) dürfen erhöhte Werte des hochsensitiven kardialen Troponins (hs-cTn) erwartet werden. Diese Patienten sind von einer erhöhten Morbidität und Mortalität bedroht, die häufig auf eine kardiale Ursache zurückzuführen ist. Daher bedarf es einer adäquaten Aufarbeitung der zugrundeliegenden Ursache, die nur im Team aus Kardiologen und Neurologen gelingen kann. Da die Ursachen vielfältig, in ihrer klinischen Präsentation beim Patienten mit AIS atypisch oder stumm und einige wie ein begleitender Myokardinfarkt akut lebensbedrohlich sein können, sollte die Abklärung einem standardisierten Algorithmus zur Differenzialdiagnostik folgen. Die überwiegende Zahl der hs-cTn-Erhöhungen wird durch nichtischämische Myokardschäden im Zusammenhang mit dem AIS verursacht. Dieser Artikel stellt einen praxisorientierten Ansatz zur Differenzialdiagnostik mit dem Update des *Mannheimer Algorithmus zu akutem ischämischem Schlaganfall und Troponinerhöhung* vor.

## Abstract (en)

Elevated high-sensitivity cardiac troponin (hs-cTn) levels should be expected in about half of all patients with acute ischemic stroke (AIS). Since those patients are at risk of increased morbidity and mortality, often attributable to cardiac causes, an adequate work-up of the underlying etiology is required. This can only be achieved by a team of cardiologists and neurologists. Since underlying causes of hs-cTn elevation in AIS patients are diverse, often atypical or silent in their clinical presentation and some, such as an accompanying myocardial infarction, can be acutely life-threatening, the work-up should follow a standardized clinical algorithm. The vast majority of hs-cTn elevations are caused by non-ischemic myocardial injury associated with AIS. This work presents a practice-oriented approach to differential diagnosis with the update of the *Mannheim clinical algorithm for acute ischemic stroke and troponin elevation*.

Ein Anteil von 30 bis 60 % aller Patienten mit akutem ischämischem Schlaganfall (AIS) weist zum Diagnosezeitpunkt eine Erhöhung des hochsensitiven kardialen Troponins (hs-cTn) über dem 99. Perzentil der Allgemeinbevölkerung auf [35, 36]; hs-cTn ist ein laborchemischer Marker der Myokardzellnekrose. Nur für eine Minderheit dieser Myokardschädigungen ist bei AIS-Patienten eine Myokardinfarkt(MI)-assoziierte vulnerable Koronarplaque (sogenannte „culprit lesion“) verantwortlich [26, 31]. Im Gegensatz dazu liegt bei Patienten mit einem Nicht-ST-Strecken-Hebungs-MI („non-ST-elevation myocardial infarction“ [NSTEMI]) ohne AIS in 80 % der Fälle eine „culprit lesion“ vor [31]. Dennoch ist eine hs-cTn-Erhöhung bei AIS-Patienten ein signifikanter Prädiktor für ein schlechteres funktionelles Outcome und eine erhöhte Mortalität [2, 39], die häufig kardiovaskulär bedingt ist [7, 37].

Eine hs-cTn-Erhöhung ist bei AIS ein signifikanter Prädiktor für ein schlechteres Outcome

Die Wahrscheinlichkeit für akute kardiovaskuläre Ereignisse nach AIS ist unter den Geschlechtern vergleichbar [38]. Aus diesen Besonderheiten ergibt sich das Erfordernis einer gründlichen Abklärung des kardialen Aspekts, dem regelhaft nicht ein akutes Koronarsyndrom („acute coronary syndrome“ [ACS]) zugrunde liegt. Kriterien der ACS-Leitlinie der Europäischen Gesellschaft für Kardiologie (European Society of Cardiology [ESC]; [8]) können somit nicht unkritisch auf alle AIS-Patienten mit Erhöhung des hs-cTn-Werts angewendet werden, da unter anderem hs-cTn-Cut-off-Wert und -Dynamik für diese Situation nicht wissenschaftlich validiert sind. Die in dieser Patientengruppe nicht triviale Einordnung der hs-cTn-Erhöhung folgt der Systematik, dass sie *A. Ausdruck eines relevanten zerebralen Ischämieareals mit ausgeprägtem Stresssignal entlang der Hirn-Herz-Achse (auch als „stroke-induced heart injury“ [SIHI] bezeichnet) sein kann *oder *B. Ausdruck einer vorbestehenden (bislang vielleicht subklinischen) strukturellen Herzerkrankung, die durch* *den Stressor AIS oder im Zusammenhang mit diesem klinisch apparent wird *[4, 39].

In seltenen Fällen des Typs B liegt die Koinzidenz eines AIS und eines MI mit Ruptur oder Erosion einer koronaren Plaque (Typ-1-MI) vor [8, 26, 42]. Es ist daraus ableitbar, dass es eines differenzierten und dennoch auf der Stroke Unit praktisch handhabbaren Algorithmus bedarf, um diejenigen Patienten zu identifizieren, die in der Akutphase nach AIS einer invasiven Koronarangiographie (IKA) mit Koronarintervention bedürfen – unter Umständen unter Inkaufnahme eines erhöhten Blutungs- oder periprozeduralen Risikos wie der Präzipitation eines Delirs [14]. Mit dem Algorithmus muss es zudem möglich sein, auch AIS-Patienten ohne MI einer rationalen Diagnostik zuzuführen, um relevante kardiale Begleiterkrankungen sensitiv erkennen und individuell behandeln zu können [21]. Eine spezifische Leitlinie für diese Situation existiert aufgrund fehlender Daten aus randomisierten, kontrollierten Studien bislang nicht. Einen praxisorientierten Ansatz zur Differenzialdiagnostik stellen wir hier mit dem ersten Update des *Mannheimer Algorithmus zu akutem ischämischem Schlaganfall und Troponinerhöhung* vor ([14]; Abb. 1).

**Abb. 1:**
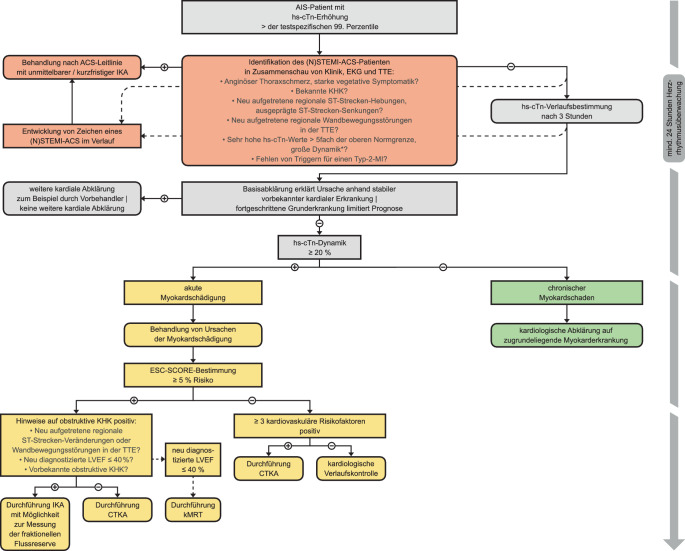
Mannheimer Algorithmus zur Differenzialdiagnostik bei AIS und Troponinerhöhung. * Bei klinischer Wahrscheinlichkeit für ein NSTEMI-ACS Verlaufsbestimmung des hs-cTn-Werts je nach lokal verfügbarem Assay bereits nach 1 oder 2 h. Invasives Vorgehen im Fall eines (N)STEMI-ACS so früh wie durch die Nutzen-Risiko-Abwägung des Teams aus Kardiologen und Neurologen vertretbar; detaillierte Erläuterungen zum Vorgehen im Text. *ACS* „acute coronary syndrome“ (akutes Koronarsyndrom), *AIS* akuter ischämischer Schlaganfall, *CTKA* Computertomographie-Koronarangiographie, *EKG* Elektrokardiogramm, *ESC* „European Society of Cardiology“ (Europäische Gesellschaft für Kardiologie), *hs-cTn* „high-sensitivity cardiac troponin“ (hochsensitives kardiales Troponin), *IKA* invasive Koronarangiographie, *KHK* koronare Herzkrankheit, *kMRT* kardiale Magnetresonanztomographie, *LVEF* linksventrikuläre Ejektionsfraktion, *MI* Myokardinfarkt, *(N)STEMI* „(non‑)ST-elevation myocardial infarction“ ([Nicht-]ST-Strecken-Hebungs-Myokardinfarkt), *TTE* transthorakale Echokardiographie

## Klassifikation der Ursache der Myokardschädigung

Beim Patienten mit ACS ist der Grund der ärztlichen Konsultation regelhaft eine akute Verschlechterung der kardialen Belastbarkeit, beispielsweise durch anginöse Thoraxschmerzen, Dyspnoe oder atypische Beschwerden. In der Folge wird zwischen „ST-elevation myocardial infarction“ (ST-Strecken-Hebungs-MI [STEMI]), NSTEMI, einer instabilen Angina pectoris oder einer Differenzialdiagnose zum ACS unterschieden, wobei auch der hs-cTn-Wert herangezogen wird [8]. Jene Patienten werden primär in kardiologischen Fachabteilungen behandelt. Der Auftrag an das Team der Stroke Unit ist davon abweichend, eine etwaige Erhöhung des leitliniengerecht im Rahmen der AIS-Aufnahmeroutine bestimmten hs-cTn-Werts (Klasse-I-Empfehlung) ex post zu interpretieren [17].

### Identifikation von Patienten mit begleitendem (N)STEMI-ACS

Initial ist die Identifikation des AIS-Patienten mit begleitendem (N)STEMI-ACS und Typ-1-MI (in der Gesamtheit seltene, aber klinisch dennoch auftretende Konstellation [26, 28]) erforderlich, da dann bei hohem oder sehr hohem Risiko für Periinfarktkomplikationen eine unmittelbare bzw. binnen 24 h nach Erstkontakt durchgeführte IKA mit Interventionsbereitschaft notwendig ist (Tab. 1). Die sichere Identifikation eines Patienten mit (N)STEMI-ACS im Kontext einer anderen Akuterkrankung ist in der klinischen Praxis nicht immer einfach und bedarf einer umfangreichen klinisch-kardiologischen Expertise. Dies wird auch von der ESC-Leitlinienkommission anerkannt [8]. Ein (N)STEMI-ACS kann sowohl über den Mechanismus eines Typ-1-MI als auch bedingt durch eine relative Imbalance von Sauerstoffangebot und -bedarf am Myokard ohne Ruptur oder Erosion einer Koronarplaque (Typ-2-MI) entstehen. Diese Differenzierung ist selbst für erfahrene Kardiologen nicht trivial [19, 34]. Sie ist aber klinisch bedeutsam, da ein Typ-2-MI, der ebenso mit einer erhöhten Mortalität einhergeht, regelhaft keine unmittelbare Koronarintervention erfordert, insofern keine instabile Koronarplaque vorliegt [12, 42]. Vielmehr ist initial eine Behandlung der zugrundeliegenden Ursache erforderlich (Tab. 2).

Der absolute hs-cTn-Wert ist allein kein ausreichender Prädiktor, um einen Typ-1-MI voherzusagen

Die absolute Höhe des hs-cTn-Werts ist kein ausreichender alleiniger Prädiktor, um einen Typ-1-MI abzugrenzen [31, 34]. Die Identifikation von (N)STEMI-ACS-Patienten mit Typ-1-MI gründet sich auf die Würdigung der klinischen Präsentation, die Interpretation von Veränderungen im Elektrokardiogramm (EKG), die klinische Untersuchung, die Berücksichtigung von Begleit- und Vorerkrankungen sowie eventuell auch eine Point-of-care-Echokardiographie [8]. Auf einen Typ-1-MI weisen dabei folgende Punkte hin, insbesondere wenn sie in Kombination vorliegen [8, 11]:

- Anginöse Thoraxschmerzen oder eine starke vegetative Symptomatik
- Bekannte koronare Herzkrankheit (KHK)
- (Neu aufgetretene) regionale ST-Strecken-Hebungen am J-Punkt in ≥ zwei benachbarten Ableitungen (unter Berücksichtigung von V7 bis V9 und V3R/V4R), ausgeprägte ST-Strecken-Senkungen oder ein kompletter Schenkelblock (mit starker Symptomatik myokardialer Ischämie)
- (Neu aufgetretene) regionale Wandbewegungsstörungen in der Echokardiographie
- Sehr hohe hs-cTn-Werte (> 5faches der oberen Normgrenze) oder große Dynamik des hs-cTn-Werts in der seriellen Messung
- Fehlen von Triggern für einen Typ-2-MI (vergleiche Tab. 2)

**Tab. 1:** Sehr hohes bzw. hohes Risiko für Periinfarktkomplikationen, verbunden mit der Indikation zur unmittelbaren invasiven Koronarangiographie bzw. invasiven Koronarangiographie binnen 24 h [8]

*Sehr hohes Risiko*
ST-Strecken-Hebungs-Myokardinfarkt
Hämodynamische Instabilität oder kardiogener Schock
Wiederkehrender oder therapierefraktärer Thoraxschmerz
Akute Herzinsuffizienz im Zusammenhang mit anhaltender myokardialer Ischämie
Lebensbedrohliche Arrhythmie oder Herzstillstand nach Krankenhausaufnahme
Mechanische Komplikation (Ruptur der freien Wand, des interventrikulären Septums oder eines Papillarmuskels mit konsekutiver akuter Herzinsuffizienz)
Wiederkehrende dynamische EKG-Veränderungen, die auf eine Ischämie hinweisen (insbesondere mit wiederkehrenden ST-Strecken-Hebungen)
*Hohes Risiko*
Dynamische ST-Strecken- oder T‑Wellen-Veränderungen
Transiente ST-Strecken-Hebungen (≤ 20 min)
GRACE-Risikoscore ≥ 140 Punkte

*EKG* Elektrokardiogramm, *GRACE* Global Registry for Acute Coronary Events

**Tab. 2:** Ursachen eines Typ-2-Myokardinfarkts [9, 10, 13, 43]

Akute Herzinsuffizienz
(Schwere) Anämie
Dekompensierte Aortenklappenstenose
Arrhythmie
(Schwere) arterielle Hypertonie
(Schwere) arterielle Hypotonie
Hypoxie
Koronardissektion
Koronare Embolie
Koronarspasmus, koronare mikrovaskuläre Dysfunktion (auch im Rahmen eines Takotsubo-Syndroms)
(Ausgeprägte) linksventrikuläre Hypertrophie
Sepsis

Neben dem Risiko, den (N)STEMI-ACS-Patienten nicht zu erkennen, besteht das diametrale Risiko einer zu großzügigen Zuführung von Patienten zur IKA mit möglicherweise folgender Intervention einer stabilen Koronarstenose, die als „culprit lesion“ eines ACS fehlinterpretiert wird [12]. Die weitere Diagnostik und Therapie unterscheiden sich beim (N)STEMI-ACS-Patienten mit AIS grundsätzlich nicht von allgemeinen Standards und es kommt die unlängst publizierte ESC-Leitlinie zur Anwendung [8]. Die Behandlung erfolgt jedoch nach Nutzen-Risiko-Abwägung innerhalb des Teams aus Kardiologen und Neurologen. Hierbei zu beachten ist die Empfehlung zum limitierten Einsatz von Prasugrel, da es bei AIS-Patienten aufgrund des erhöhten Blutungsrisikos kontraindiziert ist [3].

### Myokardschädigung ohne Hinweis auf (N)STEMI-ACS

In der überwiegenden Zahl der Fälle kann die hs-cTn-Erhöhung nicht mit einem (N)STEMI-ACS erklärt werden [26, 29, 31]. Der Fokus muss dann darauf liegen, mittels rationaler und kosteneffektiver Diagnostik die Ursache der Myokardschädigung aufzudecken, um akute oder chronische Myokarderkrankungen zu differenzieren, optimal zu behandeln und damit die Prognose des Patienten zu verbessern. Bei der *akuten Myokardschädigung* kommen ischämische Mechanismen des Typ-2-MI im Zusammenhang mit der Stressreaktion durch den AIS ebenso infrage wie nichtischämische, rein neurogene SIHI-Mechanismen (akuter Myokardschaden; Ursachen in Tab. 3) bis zum Vollbild des Takotsubo-Syndroms [37].

**Tab. 3:** Ursachen eines akuten Myokardschadens [9, 10, 13]

Kardial	Nichtkardial
Akute Herzinsuffizienz	(Starke körperliche) Anstrengung
Arrhythmie	Chemotherapie, Strahlentherapie
Defibrillation/Kardioversion	(Schwere systemische) Infektion, Sepsis
Kardialer Eingriff	Lungenarterienembolie
Kardiale Kontusion	Niereninsuffizienz
Myokarditis	Pulmonale Hypertonie
Takotsubo-Syndrom	„Stroke-induced heart injury“

Ein *chronischer Myokardschaden* ist bei annähernd 85 % der AIS-Patienten für die hs-cTn-Erhöhung verantwortlich [36]. Hierbei spielen insbesondere bislang inapparente Kardiomyopathien, aber auch Endo- oder (Peri‑)Myokarditiden eine Rolle, die wiederum Ursache eines kardioembolischen AIS sein können [24]. Eine KHK mit ≥ 50 %iger Stenose in mindestens einer epikardialen Koronararterie liegt insgesamt bei etwa einem Drittel der AIS-Patienten vor [18]. Akute Myokardschädigungen führen gegenüber chronischen Myokardschäden bei AIS-Patienten zu einer höheren Krankenhaussterblichkeit [35].

Im ersten Schritt dieser weiteren Differenzierung sollte dichotom zwischen akuter (nach der vierten universellen Definition des MI definiert als Positiv- oder Negativdynamik des hs-cTn-Werts von ≥ 20 % binnen eines 3 h-Intervalls) und chronischem Myokardschaden unterschieden werden [42]. Das 3 h-Messintervall des hs-cTn-Werts ist nicht mit dem 0/1(/2) h-Intervall zum Ein- oder Ausschluss im ACS-Algorithmus gleichzusetzen, der vorrangig einer schnellen Diagnosestellung beim MI-Patienten dient [8]. Wir führen daher bei allen AIS-Patienten mit hs-cTn-Erhöhung ohne Hinweise auf ein ACS auch weiterhin eine Verlaufsbestimmung nach exakt 3 h durch, was überdies angesichts der Initialmaßnahmen nach AIS (intravenöse Thrombolyse und/oder interventionelle Thrombektomie) als pragmatisches Zeitfenster erscheint.

Die Basisabklärung besteht aus der Erfassung vorausgehender kardialer Beschwerden (eigen- oder fremdanamnestisch), kardialer Vorbefunde und relevanter Begleiterkrankungen, einem EKG sowie einer Standardechokardiographie binnen 24 h. Wie im Abschnitt „Praktisches Vorgehen in der weiteren Differenzialdiagnostik“ ausgeführt, können durch diese Schlüsselmaßnahmen schon erste Rückschlüsse auf die Ursache der Myokardschädigung und die im Weiteren erforderlichen Maßnahmen abgeleitet werden.

Treten in diesem Verlauf Zeichen eines ACS auf, wird umgehend auf den ACS-Algorithmus der ESC gewechselt. Zahlreiche AIS-Patienten können aufgrund von neurologischen Symptomen wie Aphasie, Dysarthrie, Vigilanzminderung, Neglect/Anosognosie oder schlaganfallassoziiertem Delir ACS-Symptome nicht äußern oder sind in deren Perzeption eingeschränkt [6]. Zur sicheren Identifikation des Patienten mit AIS-(N)STEMI-ACS sollte in diesem Stadium der Observanz auf dynamische EKG-Veränderungen, unerwartete hämodynamische Instabilität oder Rhythmusinstabilität geachtet werden. Derart sollten auch Patienten mit Koronardissektion, Koronarembolie und Koronarspasmus identifiziert werden können; diese Erkrankungen werden dem Typ-2-MI-Mechanismus zugeordnet, stellen allerdings ebenso akut und interventionell zu therapierende Entitäten dar [8, 42].

## Erweiterte Diagnostik auf eine strukturelle Herzerkrankung bei akuter und chronischer Myokardschädigung

Die weiterführende Diagnostik wird nach klinischer Stabilisierung des AIS, aber im zeitlichen Zusammenhang mit dem Indexaufenthalt auf der Stroke Unit durchgeführt. Falls in der Basisabklärung keine abschließende Erklärung für die hs-cTn-Erhöhung gefunden werden kann, kommen verschiedene bildgebende Verfahren in Betracht, um eine bisher klinisch inapparente strukturelle Herzerkrankung aufzudecken oder auszuschließen.

### Computertomographische und invasive Koronarangiographie

In der DISCHARGE-Studie konnte bei Patienten mit einer stabilen Angina pectoris und einer mittleren Vortestwahrscheinlichkeit für eine KHK durch die alternative Anwendung einer Computertomographie-Koronarangiographie (CTKA) im Vergleich zur IKA ein vergleichbar niedriges Risiko schwerwiegender unerwünschter kardiovaskulärer und zerebrovaskulärer Ereignisse (hier: kardiovaskulärer Tod, MI oder AIS) von je < 5 % in einem medianen Follow-up-Zeitraum von 3,5 Jahren erreicht werden [40]. Die Durchführung einer unmittelbaren CTKA bei Patienten mit klinischem Verdacht auf ein NSTEMI-ACS und intermediärem KHK-Risiko führte zu einer Reduktion von IKA und verbesserte das Verhältnis von Koronarinterventionen zu durchgeführten IKA [15]. In der CTKA-Gruppe traten binnen eines Jahres nicht mehr Typ-1-MI, Typ-4b-MI (durch Stentthrombose) oder Todesfälle auf (5,8 % bei CTKA vs. 6,1 % bei Standardprozedere). Die CTKA kann damit bei AIS-Patienten mit hs-cTn-Erhöhung und niedriger Vortestwahrscheinlichkeit einer obstruktiven KHK als sinnvoller Torwächter zur IKA verstanden werden, um unnötige, nicht mit einer Koronarintervention verbundene invasive Prozeduren und damit potenzielle prozedurale Komplikationen in dieser vulnerablen Patientenpopulation zu vermeiden [44]. Dem kommt nicht zuletzt die immer weiter verbesserte Bildakquise moderner Systeme entgegen, die bei stark kalzifizierten Koronarplaques oder intrakoronaren Stents weniger anfällig sind und mit geringerer Strahlenbelastung einhergehen [20]. Weiterhin eingeschränkt ist die Aussagekraft der CTKA bei extremer Adipositas, ausgeprägter Arrhythmie, Tachykardie oder kooperationsunfähigen Patienten [16, 25].

Die CTKA kann in einem Schritt eine obstruktive KHK als Ursache der Myokardschädigung sensitiv ausschließen sowie gleichzeitig auch seltene alternative Ursachen wie kardiale Raumforderungen aufdecken [44]. Zudem ermöglicht sie eine Risikostratifizierung nichtobstruktiver Koronarplaques mithilfe des Agatston-Scores [5]. In Evaluation befinden sich aktuell CTKA-Verfahren, welche die hämodynamische Relevanz intermediärer Koronarstenosen unmittelbar klären [5].

### Kardiale Magnetresonanztomographie

Die kardiale Magnetresonanztomographie (kMRT) ist besonders geeignet für die Feststellung inapparenter myokardialer Veränderungen und hat mit einer Belastungsuntersuchung eine hohe Sensitivität und Spezifität in der myokardialen Ischämiediagnostik [1, 27]. Die kMRT kommt daher zum Einsatz, um myokardiale Erkrankungen, wie hereditäre oder erworbene Kardiomyopathieformen, eine (Peri‑)Myokarditis oder eine kardiale Beteiligung bei Systemerkrankungen abzuklären. Durch medikamentöse Stressinduktion mit Adenosin oder Dobutamin (Belastungstest) können außerdem vermutete oder intermediär diagnostizierte Koronarstenosen auf ihre funktionelle Relevanz hin untersucht werden [25, 27]. Dabei ist zu beachten, dass bei AIS-Patienten mit KHK auch gehäuft Stenosen der A. carotis vorliegen [23]. Auch in der myokardialen Vitalitätsdiagnostik bei bekannter KHK ist die kMRT mit Bestimmung des „late gadolinium enhancement“ Mittel der Wahl [30]. Zudem liefert die kMRT wertvolle Zusatzinformationen bei der Detektion und Differenzialdiagnostik kardialer Raumforderungen (thrombotisch vs. maligne) und kann derart auch zur Aufklärung kardialer Emboliequellen bei embolischem AIS beitragen [1].

## Praktisches Vorgehen in der weiteren Differenzialdiagnostik

In einigen Fällen kann bereits durch die Basisabklärung die Ursache der Myokardschädigung herausgearbeitet werden, insbesondere bei bekannter struktureller Herzerkrankung. Durch die (Fremd-)Anamnese auf eine (sub)akute Verschlechterung der kardialen Belastbarkeit wird in dieser Situation über die Dringlichkeit einer weiteren kardialen Abklärung entschieden. Patienten mit einem fortgeschrittenen Frailty-Syndrom oder einer fortgeschrittenen onkologischen Erkrankung, die nach Einschätzung der Behandler nicht von invasiven kardiologischen Therapien in der Folge einer weiteren Abklärung profitieren würden, können von dieser weiteren Diagnostik ausgenommen werden.

### Patienten mit akuter Myokardschädigung ohne Hinweis auf ein (N)STEMI-ACS

Hier sollten die der akuten Myokardschädigung zugrundeliegenden Ursachen gemäß Tab. 2 und 3 behandelt und die Wahrscheinlichkeit eines tödlichen kardiovaskulären Ereignisses binnen 10 Jahren gemäß ESC SCORE bestimmt werden [33]. Bei einer Wahrscheinlichkeit < 5 % (Niedrigrisikogruppe) führen wir nur bei deutlich erhöhtem kardiovaskulärem Risiko (mindestens 3 Risikofaktoren, vergleiche Tab. 4) zum Ausschluss einer KHK eine CTKA mit CT-Koronarkalkscreening durch. Hier hat sich in der Praxis gezeigt, dass gehäuft nichtobstruktive Koronarplaques aufgedeckt werden können, die im Zusammenhang mit dem Gesamtrisikoprofil des Patienten gewürdigt werden. Ohne ein solches kardiovaskuläres Risikoprofil wird der Patient einer kurzfristigen ambulanten Nachsorge mit Echokardiographie unterzogen, um einen etwaigen dynamischen Prozess aufzudecken. Bei Patienten < 60 Jahren mit dieser Konstellation und ohne ausreichend erklärende Ursache in der Basisabklärung ergänzen wir eine kMRT, um etwaige inapparente Myokarderkrankungen zu detektieren (insbesondere aus dem inflammatorischen Formenkreis), die der Echokardiographie verborgen geblieben sind.

**Tab. 4:** Kardiovaskuläre Risikofaktoren

Adipositas
Alter > 70 Jahre
Arterieller Hypertonus (medikamentös behandelt)
Diabetes mellitus (medikamentös behandelt)
Dyslipoproteinämie (LDL-Cholesterin > 150 mg/dl oder medikamentös behandelt)
Positive Familienanamnese bezüglich kardiovaskulärer Erkrankung
Schädlicher Gebrauch von Tabak (aktiver Konsum oder zurückliegend ≥ 10 „pack years“)

*LDL* Low-density-Lipoprotein

Bei akuter Myokardschädigung ist die Würdigung des kardiovaskulären Risikoprofils entscheidend

Bei einer Wahrscheinlichkeit ≥ 5 % (Hochrisikogruppe) führen wir in jedem Fall eine CTKA mit CT-Koronarkalkscreening durch. In dieser Risikogruppe können nach eigener Erfahrung regelhaft Koronarstenosen < 50 % aufgedeckt werden, die eine regelmäßige ambulante kardiologische Nachsorge nach sich ziehen sollten. Liegen in der Basisabklärung dieser Hochrisikopatienten bereits Hinweise auf eine obstruktive KHK vor, führen wir eine IKA mit Möglichkeit zur Messung der fraktionellen Flussreserve vor etwaiger Revaskularisation durch, sobald dies vonseiten des zerebralen Risikos vertretbar ist [25, 32]. Zu den Hinweisen auf das Vorliegen einer obstruktiven KHK zählen eine neu aufgetretene regionale Auffälligkeit im EKG bzw. in der Echokardiographie oder eine neu diagnostizierte linksventrikuläre Ejektionsfraktion (LVEF) ≤ 40 % sowie die vorbekannte KHK (≥ 50 %ige Stenose in mindestens einer epikardialen Koronararterie oder revaskularisierte KHK). Zu beachten ist, dass der Nutzen einer verzögerten Koronarrevaskularisation > 72 h nach Myokardschädigung hinsichtlich der Verhinderung kardialer Ereignisse im Verlauf unklar ist [22]. Daher ist es uns wichtig, die Ischämierelevanz einer Koronarstenose unter anderem durch die präprozedurale Bildgebung zu erfassen. Bei der Erstdiagnose einer systolischen Herzinsuffizienz wird zudem regelhaft eine kMRT ergänzt.

### Patienten mit chronischem Myokardschaden

Die Ursache eines chronischen Myokardschadens liegt > 24 h zurück [41]. Keineswegs handelt es sich hierbei in allen Fällen um eine bereits bekannte strukturelle Herzerkrankung; auch ein mehrere Tage zurückliegender STEMI im subakuten Stadium (Stadium II) kann die formale Definition eines chronischen Myokardschadens erfüllen. In jedem Fall sollte im unmittelbaren Zusammenhang mit dem Indexaufenthalt auf der Stroke Unit eine patientenindividuelle Abklärung durch einen Kardiologen erfolgen. Eine vorausgehende Verschlechterung der kardialen Belastbarkeit, zu einer Herzinsuffizienz passende Befunde in der klinischen Untersuchung, neue regionale Auffälligkeiten im EKG oder in der Echokardiographie, eine neu diagnostizierte LVEF ≤ 40 % oder sehr hohe hs-cTn-Werte (> 5faches der oberen Normgrenze) geben Anlass zu einem frühzeitigen Vorgehen (sofern hinsichtlich des zerebralen Risikos vertretbar), gegebenenfalls noch im Rahmen des Indexaufenthalts.

## Diskussion des Mannheimer Algorithmus anhand von Fällen aus der Praxis

### Fall I: Akuter ischämischer Schlaganfall und nichtischämischer akuter Myokardschaden

Ein 65-jähriger Patient wurde mit in der Nacht aus dem Erwachen heraus akut aufgetretener sensomotorischer Hemiparese links zugewiesen. Aufgrund der initial schweren neurologischen Symptomatik habe er erst nach klinischer Besserung den Rettungsdienst verständigen können. Bei Vorstellung außerhalb des 4,5 h-Zeitfensters und fehlendem Nachweis eines akuten Verschlusses eines großen intrakraniellen Gefäßes bestand keine Option zur Schlaganfallakuttherapie mittels intravenöser Thrombolyse oder interventioneller Thrombektomie. Zum Vorstellungszeitpunkt persistierte lediglich eine Hemihypästhesie in der linken Körperhälfte (National-Institutes-of-Health-Stroke-Scale[NIHSS]-Score: 1 Punkt). In der kranialen MRT zeigte sich eine bereits in der Fluid-attenuated-inversion-recovery(FLAIR)-Sequenz demarkierte akute Ischämie im Stromgebiet der rechten A. cerebri media kortikal und parasagittal postzentral rechts.

Im Aufnahmelabor war der hs-cTn-I-Wert mit 0,081 µg/l (Normwert ≤ 0,045 µg/l) erhöht, in der Verlaufskontrolle nach 3 h lag er bei 0,154 µg/l (≥ 20 %iger Anstieg). Symptome einer verschlechterten kardialen Belastbarkeit oder ischämietypische EKG-Veränderungen lagen nicht vor. Kardiale Vorbefunde bestanden nicht. Die Echokardiographie zeigte eine normale LVEF ohne regionale Wandbewegungsstörungen. Die Wahrscheinlichkeit für ein tödliches kardiovaskuläres Ereignis lag gemäß ESC SCORE bei 6 % (Hochrisikogruppe).

**Abb. 2:**
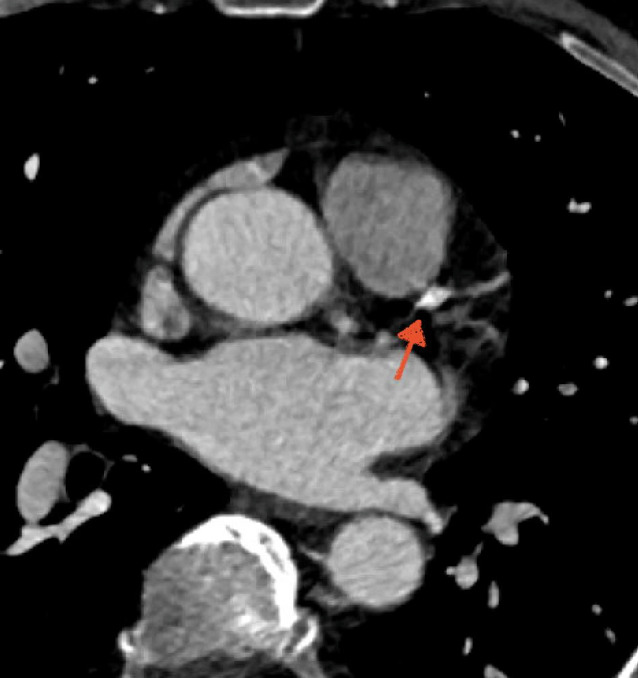
Koronare Computertomographie aus Fall I. Beispielhafte Darstellung einer kalziumreichen Koronarplaque im mittleren Ramus interventricularis anterior (*Pfeil*)

Mittels CTKA wurde eine obstruktive KHK ausgeschlossen. Es fanden sich geringgradige Koronarplaques (< 25 %) in allen großen epikardialen Koronararterien (Abb. 2). Das CT-Koronarkalkscreening ergab ein nur leicht erhöhtes relatives Risiko für ein kardiales Ereignis (52. Perzentil).

Dieser Fall demonstriert, dass eine dynamische hs-cTn-Erhöhung im Rahmen eines AIS nicht zwangsläufig auf eine obstruktive KHK oder gar einen MI zurückzuführen ist. Dank Durchführung der CTKA konnte eine IKA mit möglichen Interventionsrisiken vermieden werden. Vielmehr lag die Myokardschädigung letztlich am ehesten in den beschriebenen Mechanismen der Hirn-Herz-Achse begründet, die einen nichtischämischen akuten Myokardschaden im Sinne eines SIHI verursacht hatten.

### Fall II: Akuter ischämischer Schlaganfall und Myokarditis bei vorbestehender Kardiomyopathie

Ein 59-jähriger Patient wurde mit akut aufgetretener, seit zwei Stunden bestehender Aphasie zugewiesen (NIHSS-Score: 6 Punkte). In der kranialen CT zeigte sich eine beginnende Ischämiedemarkation im hinteren Mediastromgebiet links. Es erfolgte eine intravenöse Thrombolyse.

Der hs-cTn-I-Wert war bei Aufnahme deutlich erhöht (5,23 µg/l; Normwert ≤ 0,045 µg/l) und stieg in der Verlaufskontrolle nach 3 h weiter an (6,36 µg/l; ≥ 20 %iger Anstieg). Symptome einer verschlechterten kardialen Belastbarkeit oder ischämietypische EKG-Veränderungen bestanden nicht. Fremdanamnestisch lag eine systolische Herzinsuffizienz unklarer Ätiologie vor. Eine KHK sei vor etlichen Jahren „ausgeschlossen“ worden. Allerdings war die Eigenanamnese erheblich durch die Aphasie eingeschränkt und es konnten nur rudimentäre, ältere kardiale Befunde über die Hausarztpraxis eingeholt werden. Die Wahrscheinlichkeit für ein tödliches kardiovaskuläres Ereignis binnen 10 Jahren lag gemäß ESC SCORE bei 2 % (Niedrigrisikogruppe). Die Echokardiographie zeigte eine mittelgradig eingeschränkte LVEF (etwa 35 %) ohne regionale Wandbewegungsstörungen.

**Abb. 3:**
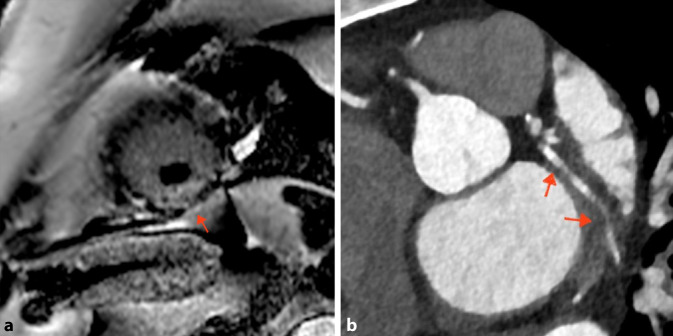
Kardiale Magnetresonanztomographie und Computertomographie-Koronarangiographie aus Fall II. **a** Subendokardiales „late gadolinium enhancement“ im Versorgungsgebiet des Ramus circumflexus (*Pfeil*). **b** Koronarplaques exemplarisch im Ramus circumflexus (*Pfeile*)

Zur Klärung der Kardiomyopathieätiologie und unter der Angabe, dass zurückliegend eine obstruktive KHK ausgeschlossen worden war, wurde zunächst eine kMRT durchgeführt. Hierbei zeigte sich eine subendokardiale ischämische Narbe im Versorgungsgebiet des Ramus circumflexus, ein streifiges „late gadolinium enhancement“ (Abb. 3a) als Hinweis auf eine Myokarditis sowie das Bild einer dilatativen Kardiomyopathie. Die LVEF zeigte sich in dieser Untersuchung sogar hochgradig eingeschränkt (28 %). Wegen der subendokardialen ischämiesuspekten Narbe wurde eine CTKA mit CT-Koronarkalkscreening ergänzt. Hierbei wurden Plaques des Ramus interventricularis anterior und des Ramus circumflexus (Abb. 3b) sowie eine mäßige Koronarsklerose (Agatston-Score: 170) nachgewiesen. Höhergradige Koronarstenosen mit Interventionsbedarf, eine Koronardissektion oder eine Koronarembolie konnten ausgeschlossen werden.

Dieser Fall veranschaulicht, dass selbst eine ausgeprägte dynamische hs-cTn-Erhöhung auf das > 100fache der oberen Normgrenze nicht zwingend eine IKA erforderlich macht. Die Basisabklärung erbrachte keine signifikanten klinischen Hinweise auf einen Typ-1-MI. Damit unterstreicht dieser Fall auch den Mehrwert nichtinvasiver Untersuchungstechniken in der Differenzialdiagnostik einer Myokardschädigung.

### Fall III: Akuter ischämischer Schlaganfall und Myokardinfarkt

Eine 78-jährige Patientin wurde mit akut aufgetretener Hemiparese rechts und Dysarthrie zugewiesen (NIHSS-Score: 4 Punkte); das Zeitfenster war dabei unklar. Etwa 6 h zuvor war es zu einem Sturz auf der Straße gekommen, nach dem die Patientin aber noch eigenständig nach Hause gekommen war. Dort war dann ein erneuter Sturz mit oben beschriebener Symptomatik aufgetreten und es war der Rettungsdienst verständigt worden. In der initialen kranialen MRT zeigte sich eine akute Ischämie im Bereich der Capsula interna links, am ehesten mikroangiopathischer Genese. Bei Diffusion-weighted-imaging(DWI)-FLAIR-Mismatch wurde eine intravenöse Thrombolyse durchgeführt. Anschließend kam es jedoch zu einer weiteren klinischen Verschlechterung mit Plegie des rechten Arms und Aphasie (NIHSS-Score: 12 Punkte).

Im Rahmen der Aufnahmediagnostik fiel ein erhöhter hs-cTn-I-Wert von 1,52 µg/l (Normwert ≤ 0,045 µg/l) auf, der in der Verlaufskontrolle nach 3 h auf 5,81 µg/l anstieg. Die Patientin äußerte, eingeschränkt durch die Aphasie, keine kardialen Beschwerden. Im EKG lagen deszendierende ST-Strecken-Senkungen in I sowie V3–V5 vor. Eine in der Akutsituation durchgeführte Echokardiographie ergab eine mittelgradig eingeschränkte LVEF sowie eine apikale Hypokinesie. Die Wahrscheinlichkeit für ein tödliches kardiovaskuläres Ereignis lag gemäß ESC SCORE bei 2 % (Niedrigrisikogruppe).

**Abb. 4:**
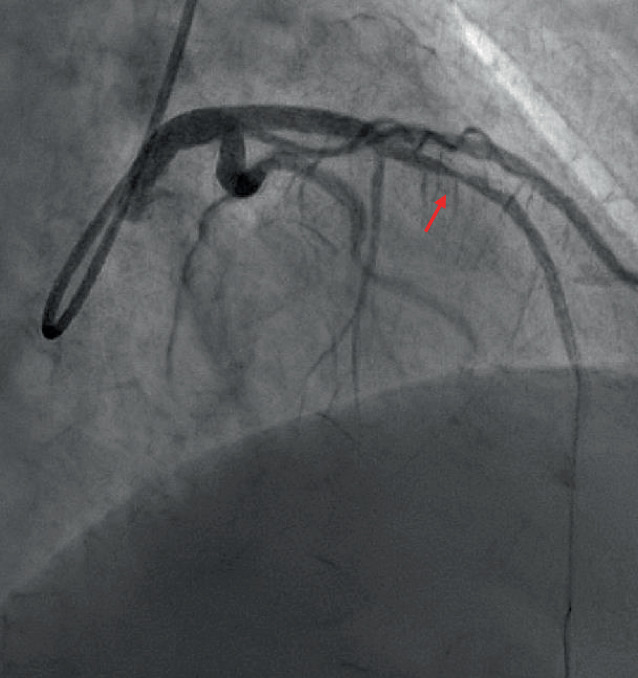
Invasive Koronarangiographie aus Fall III. Visuell obstruktive Stenose des Ramus interventricularis anterior (*Pfeil*)

Aufgrund der klinischen Hinweise auf eine obstruktive KHK wurde eine IKA innerhalb von 48 h durchgeführt. Hierbei zeigte sich eine visuell obstruktive Stenose des Ramus interventricularis anterior, die mit einem „drug-eluting stent“ behandelt wurde (Abb. 4). Eine Woche später erfolgte eine erneute Echokardiographie, in der eine nur noch leichtgradig eingeschränkte LVEF (48 %) ohne Hinweis auf eine regionale Wandbewegungsstörung zu sehen war. In der Zusammenschau ergab sich das Bild eines NSTEMI-ACS, am ehesten im Rahmen eines Typ-2-MI.

Dieser Fall zeigt, dass gerade bei AIS-Patienten ein Myokardinfarkt klinisch inapparent verlaufen kann, da zum einen die AIS-Symptomatik eine kardiale Symptomatik potenziell „verdeckt“ und zum anderen Beschwerden aufgrund einer Aphasie oder Dysarthrie nicht geäußert werden können. Bei akuter Myokardschädigung sollte daher auch im Falle einer fehlenden Symptomatik schnellstmöglich mittels EKG und Echokardiographie nach Hinweisen auf eine obstruktive KHK gesucht werden, um die weitere Therapie nicht zu verzögern.

## Fazit für die Praxis

- Der prognostische Nutzen einer invasiven Koronarangiographie (IKA) abseits zwingender Indikationen (klinische Hinweise auf akutes Koronarsyndrom mit Typ-1-Myokardinfarkt, Koronardissektion, Koronarembolie, persistierender Koronarspasmus, kardiogener Schock) bleibt bei Patienten mit akutem ischämischem Schlaganfall und Erhöhung des hochsensitiven kardialen Troponins weiter unklar, sodass in der Mehrzahl der Fälle alternative Diagnostika zur ätiologischen Klärung angewendet werden können.
- Zur evidenzbasierten Strukturierung eines risikoadaptierten Vorgehens kann der *Mannheimer Algorithmus zu akutem ischämischem Schlaganfall und Troponinerhöhung* herangezogen werden.
- Die in Planung befindliche Studie „Prognostic Impact of Coronary Angiography in Patients with Acute Ischemic Stroke and Troponin Elevation“ (COAST) wird entscheidende neue Hinweise zum Stellenwert der IKA in dieser Population liefern und Einfluss auf das praktische Vorgehen haben.

## Abkürzungen

ACS: „Acute coronary syndrome“ (akutes Koronarsyndrom)
AIS: Akuter ischämischer Schlaganfall
CT(KA): Computertomographie(-Koronarangiographie)
EKG: Elektrokardiogramm
ESC: „European Society of Cardiology“ (Europäische Gesellschaft für Kardiologie)
FLAIR: „Fluid-attenuated inversion recovery“
hs-cTn: „High-sensitivity cardiac troponin“ (hochsensitives kardiales Troponin)
IKA: Invasive Koronarangiographie
KHK: Koronare Herzkrankheit
(k)MRT: (Kardiale) Magnetresonanztomographie
LVEF: Linksventrikuläre Ejektionsfraktion
MI: Myokardinfarkt
NIHSS: National Institutes of Health Stroke Scale
(N)STEMI: „(Non‑)ST-elevation myocardial infarction“ ([Nicht-]ST-Strecken-Hebungs-Myokardinfarkt)
SIHI: „Stroke-induced heart injury“ (schlaganfallinduzierter Herzschaden)
